# Supplementary material for: Direct aluminium-alloy upcycling from entire end-of life vehicles
Source: Nat Commun. 2026 Feb 13;17:2715. doi: 10.1038/s41467-026-69492-z (PMC13013578; doi:10.1038/s41467-026-69492-z)
Supplement: Supplementary file 1 — Supplementary Information [file 41467_2026_69492_MOESM1_ESM.pdf]

# Direct aluminium-alloy upcycling from complete end-of-life vehicles: Supplementary information

Patrick Krall[1], Irmgard Weißensteiner[2], Philip Aster[2], Phillip Dumitraschkewitz[1], Matheus A. Tunes[1], Thomas Kremmer[1], Sebastian Samberger[1], Bernhard Trink[3], Stefan Pogatscher[1,*].

[1]Chair of Nonferrous Metallurgy, Montanuniversität Leoben, Franz-Josef Straße 18, 8700 Leoben, Austria

[2]Christian Doppler Laboratory for Deformation-Precipitation Interactions in Aluminum Alloys, Montanuniversität Leoben, Franz-Josef Straße 18, 8700 Leoben, Austria

[3]Christian Doppler Laboratory for Advanced Aluminum Alloys, Montanuniversität Leoben, Franz-Josef Straße 18, 8700 Leoben, Austria

*Corresponding author: Stefan Pogatscher (stefan.pogatscher@unileoben.ac.at)

**Supplementary Note 1: Composition scenarios**

The three investigated alloys arise from a theoretically calculated composition that would arise from complete melting of the aluminium alloys of three different vehicle types. Their compositions and further a 6016 reference alloy are shown in **Supplementary Table 1**. The basic concept and the results for a low-iron containing last laboratory cooled (60 K/s)-material was presented in our previous work[1].

**Supplementary Table 1: Chemical composition of the investigated alloys oriented on three different vehicle types.** The ELV-mixes are based on today´s average European vehicle (EU), a US pickup-truck (PU) and an electric car (EC) with aluminium representing the balance to 100 % (Bal.).

| Alloy | Si [%] | Fe [%] | Cu [%] | Mn [%] | Mg [%] | Zn [%] | Al [%] |
|---|---|---|---|---|---|---|---|
| EU | 5.20 | 1.20 | 0.70 | 0.30 | 0.75 | 0.40 | Bal. |
| PU | 4.75 | 1.30 | 1.30 | 0.30 | 0.70 | 0.50 | Bal. |
| EC | 1.50 | 1.00 | 0.20 | 0.30 | 1.50 | 0.10 | Bal. |
| 6016 Ref | 1.06 | 0.19 | 0.05 | 0.06 | 0.33 | 0.01 | Bal. |

**Supplementary Note 2: Heat treatment and processing sequences**

For the three alloys, different sequences of heat treatment and processing were applied to the as-cast state (CS). This includes homogenizing (H), solution annealing, pre-ageing (PA), natural ageing (NA) and paint-baking (PB). A part of the samples was also pre-strained (PS) to 2 and 5 % plastic deformation. **Supplementary Table 2** gives an overview over the applied parameters, while **Supplementary Fig. 1** graphically illustrates the processing ways starting from solution annealing.

**Supplementary Table 2: Summary of the applied heat treatments.**

<table>
<tr><th>Condition</th><th>Homo-genising</th><th>Solution annealing</th><th>Pre-ageing</th><th>Natural ageing</th><th>Pre-straining</th><th>Paint-baking</th></tr>
<tr><td>CS</td><td>-</td><td>-</td><td>-</td><td>-</td><td>-</td><td>-</td></tr>
<tr><td>H</td><td rowspan="6">10 h heating 450 °C / 10 h 7 h heating 520 °C / 10 h</td><td>-</td><td>-</td><td>-</td><td>-</td><td>-</td></tr>
<tr><td>PA</td><td rowspan="5">520 °C / 10 min Water quenching</td><td>100 °C / 5 h</td><td>-</td><td>-</td><td>-</td></tr>
<tr><td>NAPB</td><td>-</td><td rowspan="4">25 °C / 14 d</td><td>-</td><td rowspan="4">180 °C / 20 min</td></tr>
<tr><td>PANAPB</td><td rowspan="3">100 °C / 5 h</td><td>-</td></tr>
<tr><td>PANA2%PSPB</td><td>2 %</td></tr>
<tr><td>PANA5%PSPB</td><td>5 %</td></tr>
</table>

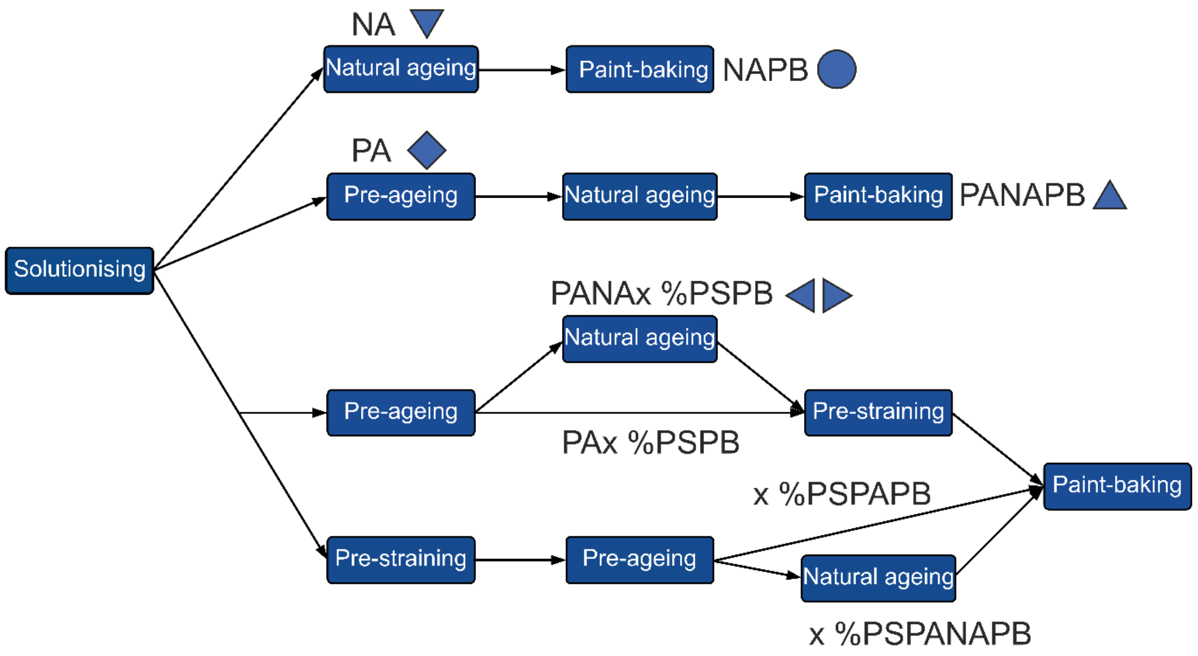


**Supplementary Fig. 1: Age hardening process.** Schematic illustration of the heat treatment procedure with and without pre-deformation and symbols indicating the processing way in **Supplementary Fig. 2**.

## Supplementary Note 3: Results of tensile testing

Tensile testing was conducted for all alloys in the conditions described in **Supplementary Fig. 1**. A general overview of the performance of the alloys compared to common automotive wrought and cast alloys is given in **Supplementary Fig. 2**. **Supplementary Fig. 3** thereby shows the results for the EU and EC-alloy in as-cast and homogenized conditions. **Supplementary Fig. 4 – Supplementary Fig. 6** show the results of the tensile tests of the different alloys after processing and further determining the strain-hardening potential on hand of Kocks-Mecking plots.

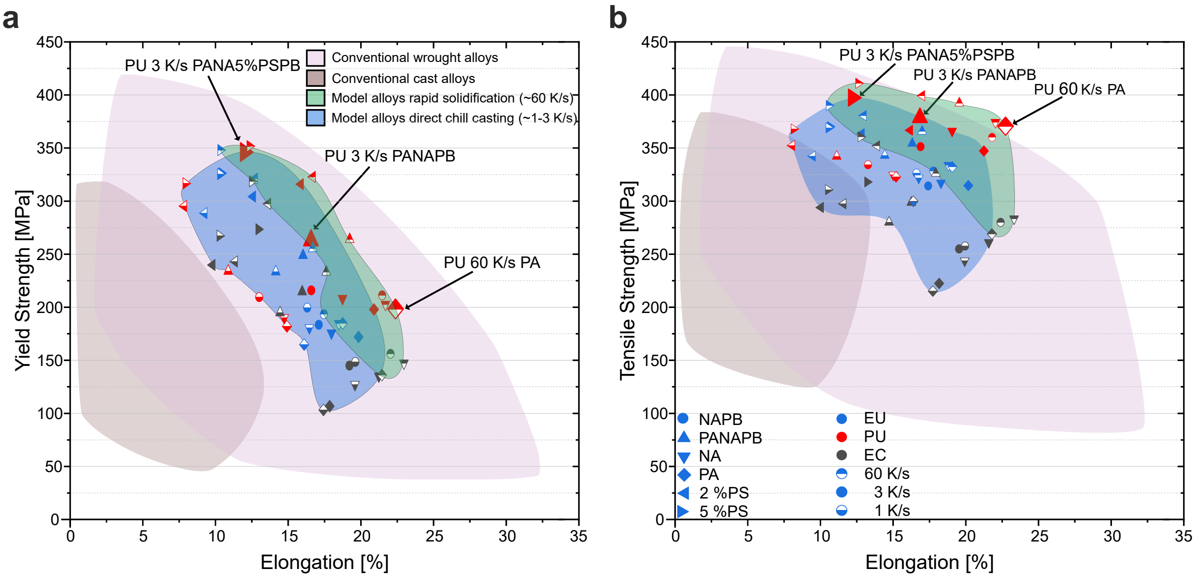


**Pre-aging (PA):** 5 h @ 100 °C **Natural aging (NA):** 14 d @ 25 °C
**Paint-baking (PB):** 20 min @ 180 °C **Pre-straining (x%PS):** includes 2 and 5 % tensile pre-deformation to PANAPB

**Supplementary Fig. 2: Strength-ductility plot comparing today's commercial automotive alloys (wrought and cast alloys) from the literature to the investigated alloys. a** Compares the yield-strengths to commercial alloys from Ansys® Granta Research Selector, Release 2025 R2, Level 3 Aero database[2] (Data reproduced courtesy of Ansys, Inc.). **b** Compares the tensile strengths to commercial alloys[2]. **a** and **b** include different cooling conditions (mimicking rapid solidification and direct chill casting)

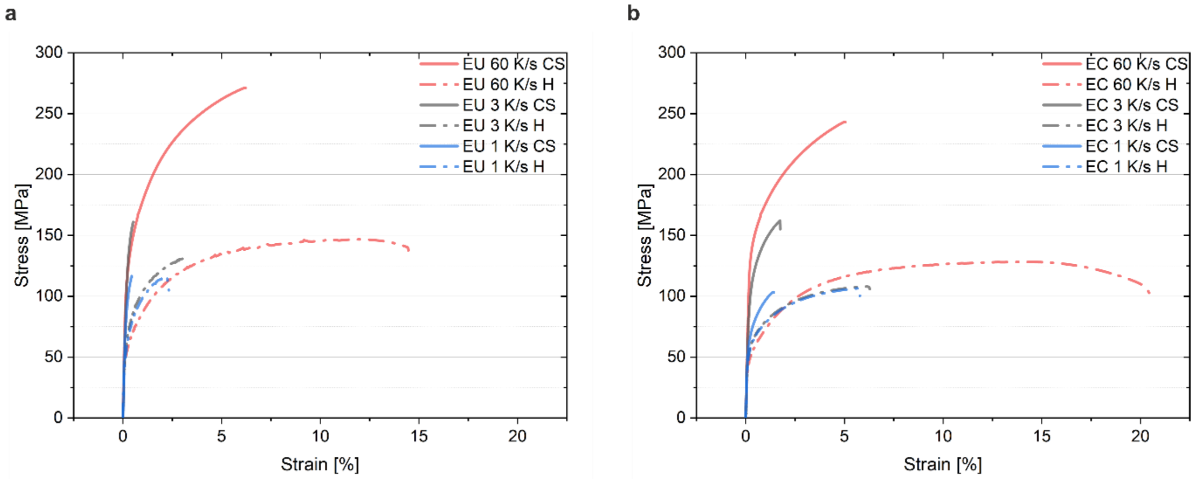


**Supplementary Fig. 3: Results of tensile tests in as-cast and homogenized conditions a** All cooling rates for alloy EU ND. **b** All cooling rates for alloy EC ND.

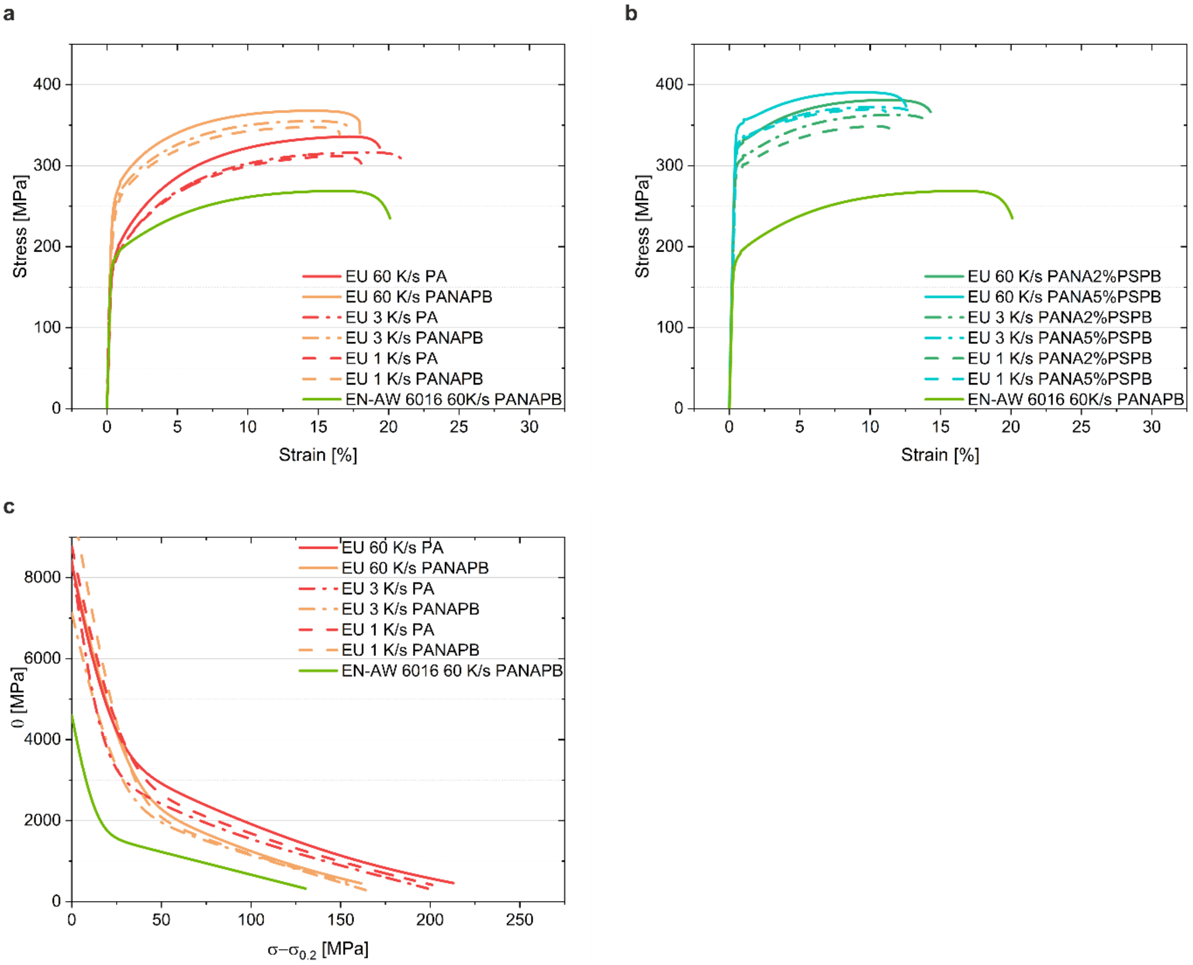


**Supplementary Fig. 4: Influence of heat treatments and pre-deformation on alloy EU ND for all cooling rates a** Results of the tensile tests for processing routes without pre-deformation in comparison to common automotive sheet alloy 6016. **b** Results of the tensile tests for processing routes including pre-deformation in comparison to common automotive sheet alloy 6016. **c** Kocks-Mecking-plots of the processing routes without pre-deformation showing higher strain-hardening potential than common automotive aluminium sheet alloy.

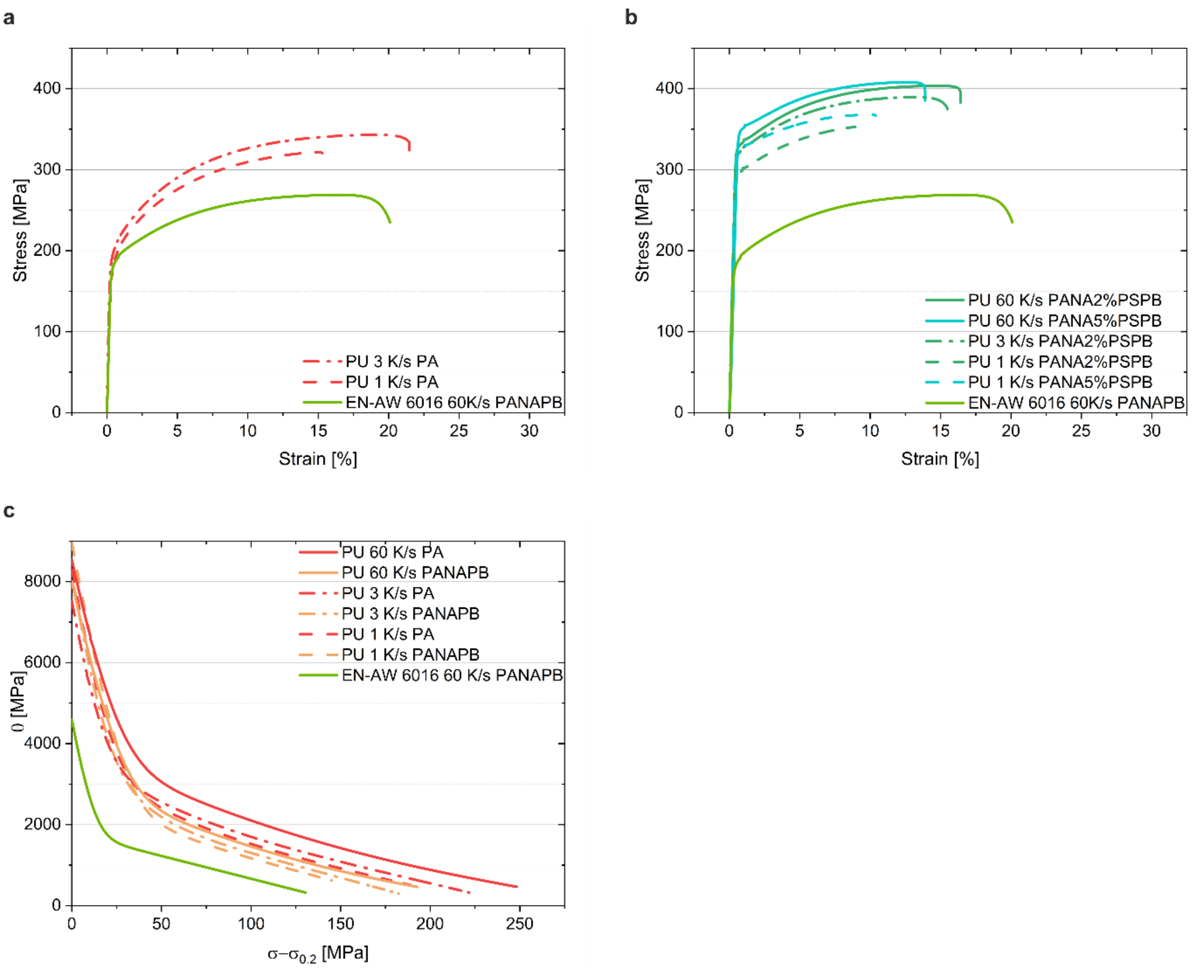


**Supplementary Fig. 5: Influence of heat treatments and pre-deformation on the remaining conditions of alloy PU ND for all cooling rates a** Results of the tensile tests for processing routes without pre-deformation in comparison to common automotive sheet alloy 6016. **b** Results of the tensile tests for processing routes including pre-deformation in comparison to common automotive sheet alloy 6016. **c** Kocks-Mecking-plots of the processing routes without pre-deformation showing higher strain-hardening potential than common automotive aluminium sheet alloy.

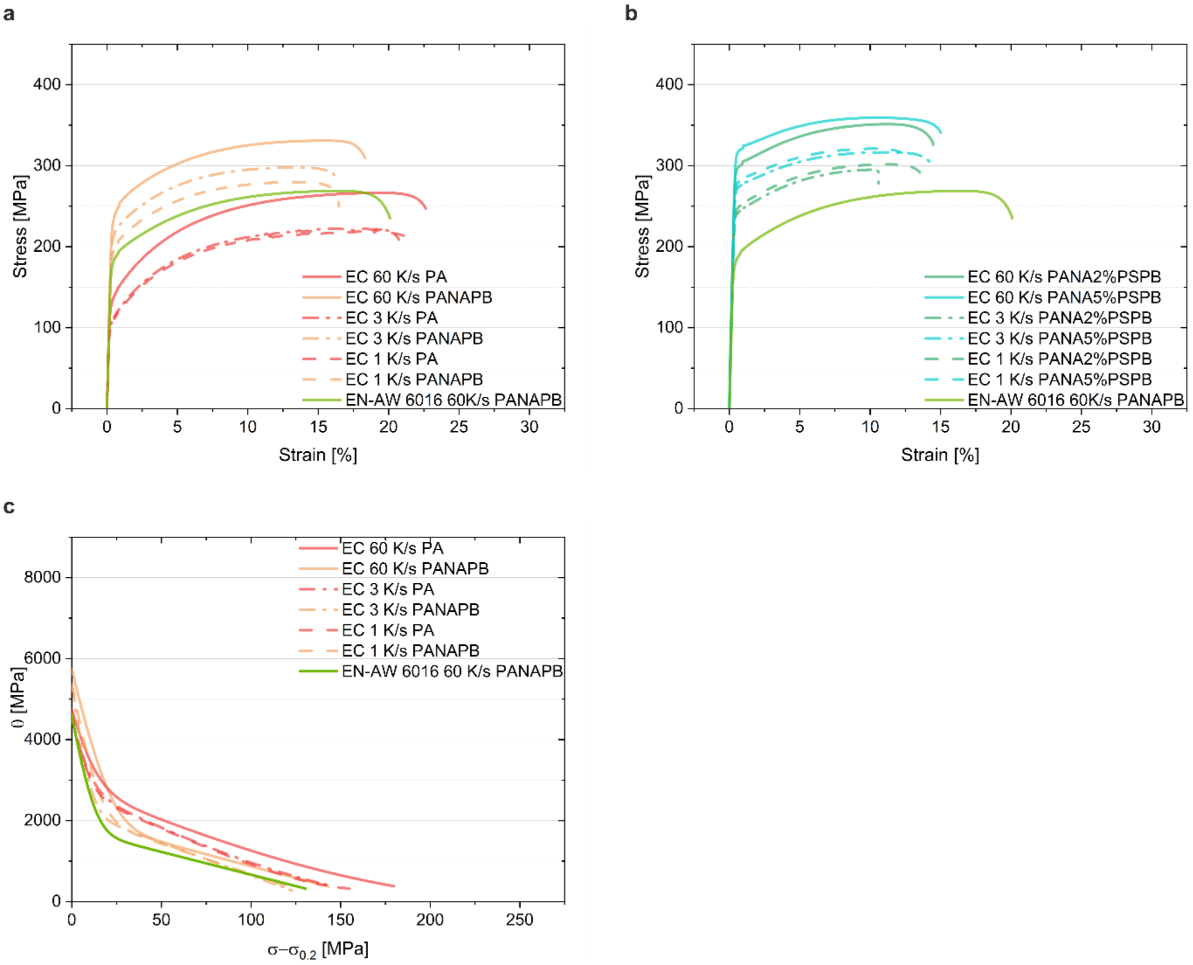


**Supplementary Fig. 6: Influence of heat treatments and pre-deformation on alloy EC ND for all cooling rate a** Results of the tensile tests for processing routes without pre-deformation in comparison to common automotive sheet alloy 6016. **b** Results of the tensile tests for processing routes including pre-deformation in comparison to common automotive sheet alloy 6016. **c** Kocks-Mecking-plots of the processing routes without pre-deformation showing higher strain-hardening potential than common automotive aluminium sheet alloy.

**Supplementary Note 4: Results of EBSD investigations**

The microscopic investigations included SEM-micrographs of the PU-alloy in all cooling conditions after 5 % pre-straining. The EBSD-mappings (**Supplementary Fig. 7**a-c) were measured after paint-baking, whilst KAM-mappings (**Supplementary Fig. 7**d-f) were taken after deformation to uniform elongation. At a cooling rate of 1 K/s, grains larger than 40 µm are prevalent, whereas at 3 K/s, fewer large grains are observed in the material (**Supplementary Fig. 7**a–c). In contrast, no grains exceeding 30 µm appear in the 60 K/s.

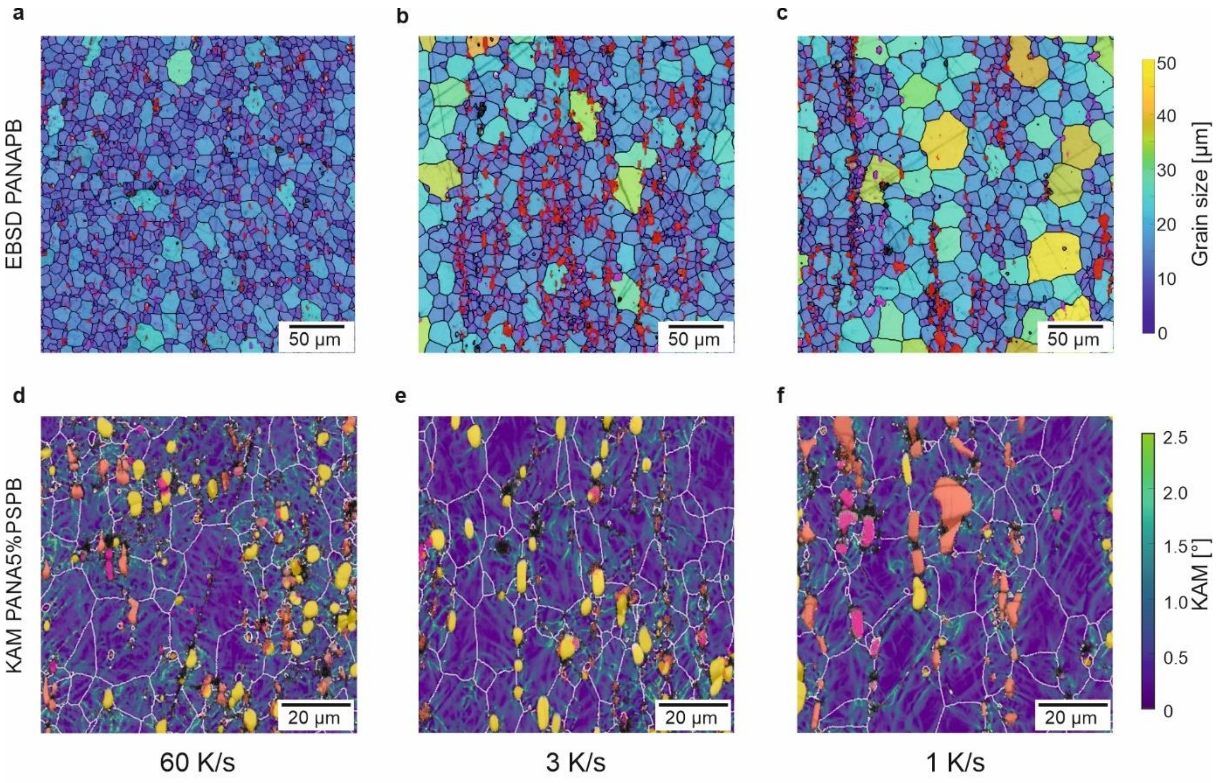


**Supplementary Fig. 7: Scanning electron microscopic investigation of the PU-alloy.** **a-c** Electron backscattered diffraction (EBSD)-mappings of the alloy PU alloy after pre-aging, natural aging and paint-bake treatment (PANAPB) following different cooling rates upon casting measured before deformation. **d-f** Kernel average misorientation (KAM)-overlay maps of the PU alloy after pre-aging, natural aging, 5 % pre-straining and paint-bake treatment (PANA5%PSPB), following different cooling rates upon casting measured at the uniform elongation. Grain boundaries are marked in white, with Si-dominant intermetallic particles (IMPs) in yellow, Fe-dominant IMPs in orange, and Mg-dominant IMPs in pink.

## Supplementary Note 5: Results of APT analysis

After PA, more than 70 % of the detected clusters contain less than 33 atoms. In PANAPB this share is 53 % and 38 % in PANA5%PSPB. In contrast, 15 % contain more than 50 atoms after PA, while 36 % and 47 % do so for PANAPB and PANA5%PSPB respectively (see **Supplementary Fig. 8**a). Regarding the Guinier-radius of the clusters and precipitates, 90 % are smaller than 2 nm in PA-condition, while for PANAPB and pre-strained states, almost 90 % have a Guinier-radius of 1–3 nm. It is conspicuous that the share of clusters and precipitates over 2 nm is larger in PANAPB than for pre-strained state (29 vs. 26 %). While the share of clusters/precipitates of more than 5 nm Guinier-radius is neglectable in PANAPB-material, in pre-strained condition it is at 2.2 % (see **Supplementary Fig. 8**b). The median Guinier radii range from 1.26 nm in the PA state to 1.61 nm after 5% pre-straining. Chemically, both the paint-baking and the pre-straining processes generate higher median magnesium concentrations (Mg/(Mg+Cu+Si+Zn) of 0.38 and 0.37, respectively, compared to 0.31 in the PA condition) (**Supplementary Fig. 8**c). An overview of the cluster chemistry in different processing conditions is given by **Supplementary Table 3**. The standard deviation of the number densities within the cluster size classes (depicted by the error bars in **Supplementary Fig. 8**a,b) is determined from the measured APT-dataset utilising a Bernoulli-approach as in equation **(1)**[3].

$$\sigma_{cluster,i} = \sqrt{\frac{C_i}{i} - \frac{{C_i}^2}{N}} \quad \mathbf{(1)}$$

$C_i$ is hereby the number of clusters of size $i$, while $N$ describes the number of atoms in the dataset.

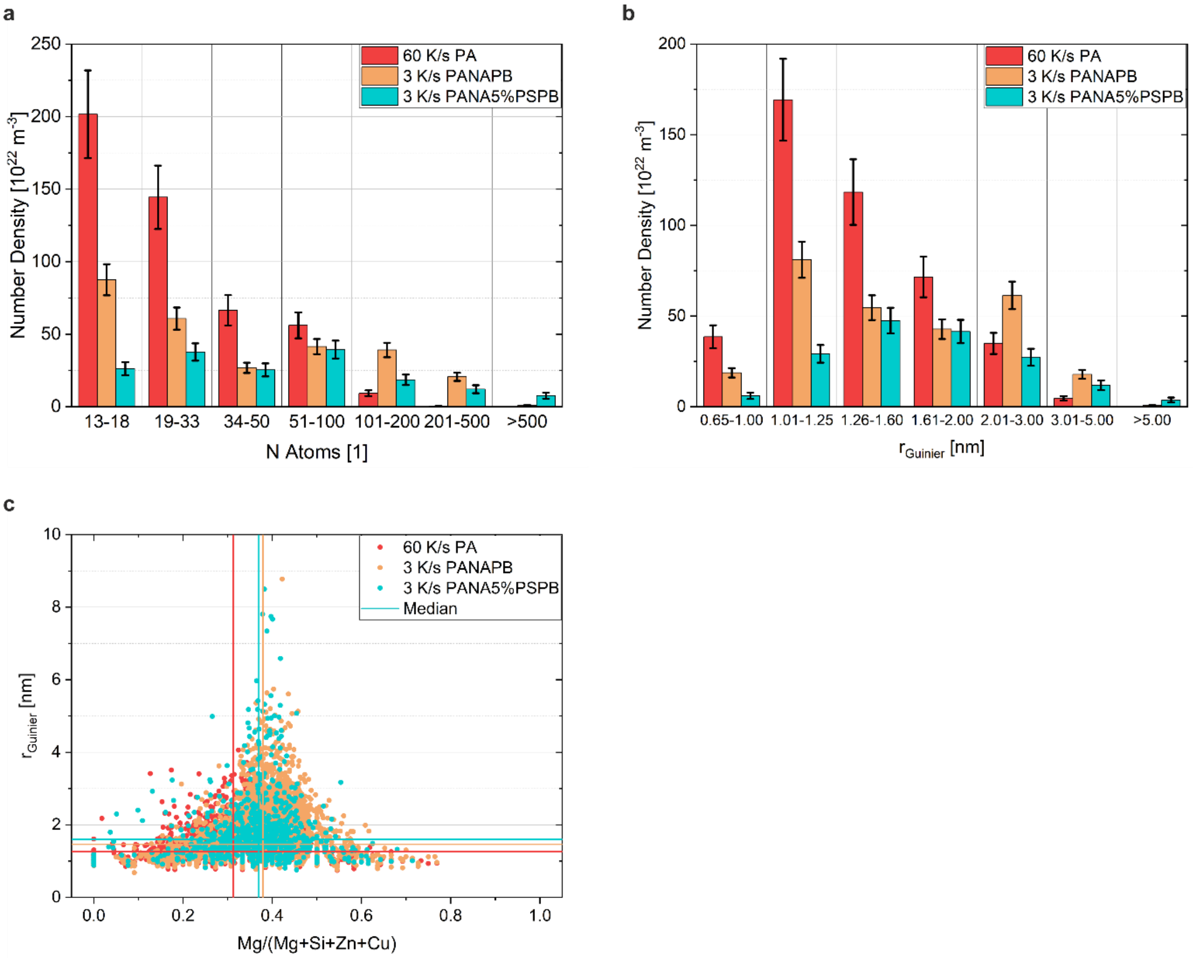


**Supplementary Fig. 8: Results of APT investigations for different processing conditions. a** Number density of clusters and precipitates over their number of atoms for 60 K/s PA, 3 K/s PANAPB and 3 K/s PANA5 %PSPB. **b** Number density of clusters and precipitates over the Guinier-radius for 60 K/s PA, 3 K/s PANAPB and 3 K/s PANA5 %PSPB. **c** Guinier-radius in dependence of the Mg-ratio in clusters and precipitates for 60 K/s PA, 3 K/s PANAPB and 3 K/s PANA5 %PSPB. The error bars indicate the standard deviation within the cluster size classes determined from the APT-dataset with a Bernoulli-approach (Eq. (**1**))[3].

**Supplementary Table 3: Chemical analysis of the clusters and precipitates for different processing conditions.**

| Alloy | Composition | Mg | Zn | Cu | Si |
|---|---|---|---|---|---|
| PU 60 K/s PA | Overall [at-%] | 0.60 ± 0.03 | 0.40 ± 0.03 | 0.52 ± 0.03 | 1.12 ± 0.03 |
| | Matrix [at-%] | 0.49 ± 0.03 | 0.40 ± 0.03 | 0.46 ± 0.02 | 0.98 ± 0.02 |
| | Cluster/Precipitates [at-%] | 2.22 ± 0.10 | 0.44 ± 0.06 | 1.29 ± 0.06 | 3.10 ± 0.06 |
| | Solute [%] | 24.58 ± 0.40 | 7.28 ± 0.64 | 16.55 ± 0.48 | 18.41 ± 0.21 |
| | Volume fraction [%] | 1.48 | | | |
| PU 3 K/s PANAPB | Overall [at-%] | 0.57 ± 0.02 | 0.37 ± 0.02 | 0.43 ± 0.02 | 1.01 ± 0.02 |
| | Matrix [at-%] | 0.37 ± 0.01 | 0.36 ± 0.02 | 0.37 ± 0.02 | 0.82 ± 0.01 |
| | Cluster/Precipitates [at-%] | 3.37 ± 0.06 | 0.43 ± 0.06 | 1.21 ± 0.06 | 3.65 ± 0.06 |
| | Solute [%] | 40.12 ± 0.24 | 8.01 ± 0.41 | 19.25 ± 0.30 | 24.69 ± 0.13 |
| | Volume fraction [%] | 2.34 | | | |
| PU 3 K/s PANA5%PSPB | Overall [at-%] | 0.59 ± 0.04 | 0.33 ± 0.04 | 0.47 ± 0.04 | 1.12 ± 0.04 |
| | Matrix [at-%] | 0.19 ± 0.02 | 0.32 ± 0.04 | 0.32 ± 0.03 | 0.73 ± 0.03 |
| | Cluster/Precipitates [at-%] | 3.54 ± 0.10 | 0.41 ± 0.11 | 1.57 ± 0.10 | 3.92 ± 0.10 |
| | Solute [%] | 72.21 ± 0.46 | 14.95 ± 0.89 | 40.34 ± 0.64 | 42.35 ± 0.24 |
| | Volume fraction [%] | 4.63 | | | |

## Supplementary Note 6: TEM Investigation

TEM-investigations reveal low dislocation density in the 60 K/s PA-sample (**Supplementary Fig. 9**a), and 3 K/s PANAPB material in **Supplementary Fig. 9**b compared to the pre-strained material in **Fig. 3**h.

For the confirmation of the Q'-hardening precipitates, the diffraction pattern of the PANA5 %PSPB was analysed and reconstructed as shown in **Supplementary Fig. 9**c and in literature [4].

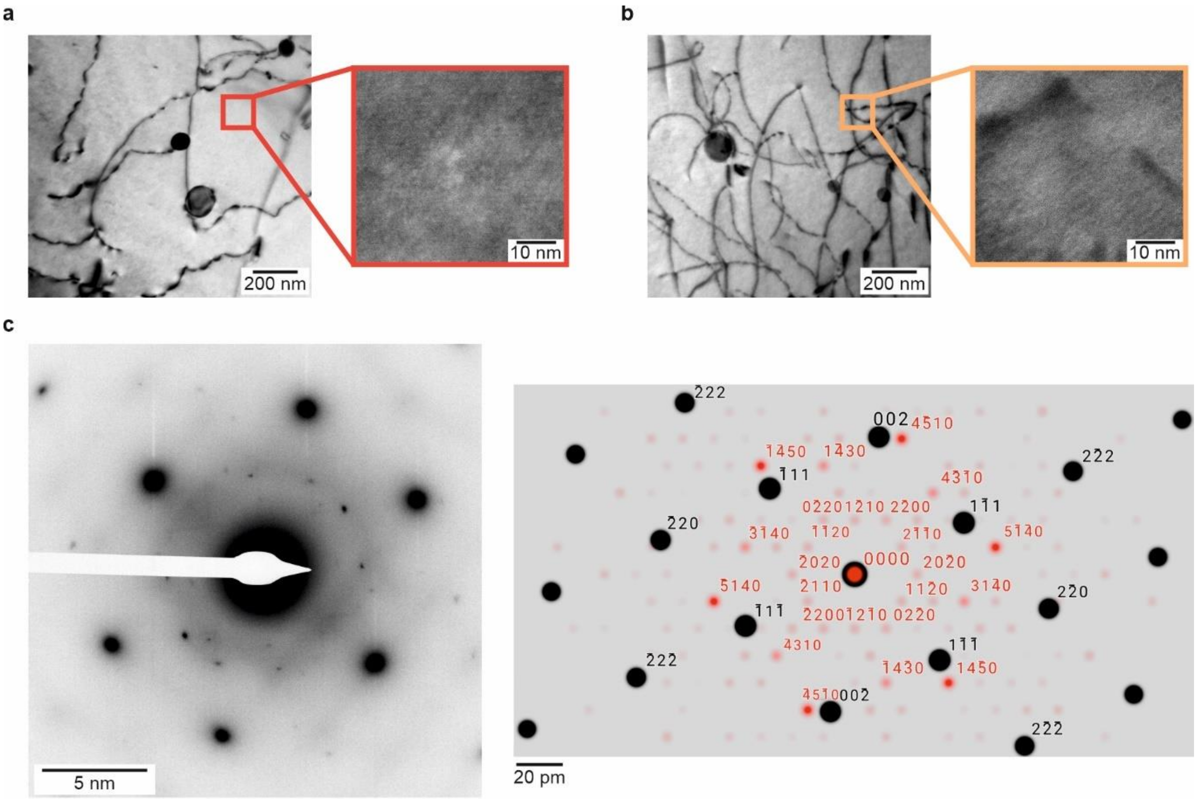


**Supplementary Fig. 9: TEM images showing interactions of dislocations and particles. (a)** In the 60 K/s PA-material with high-resolution-insert. (b) For the 3 K/s PANAPB-sample with high-resolution-image inserted. **c** Diffraction pattern and indexing of the hardening precipitates in PANA5 %PSPB-material for confirmation of Q'-phase according to literature [4].

**Supplementary Note 7: Simulation of vacancy concentration, dislocation density and diffusion ways during pre-straining**

According to Militzer et al.[5] and Robson[6] we calculate the initial ($c_0$, equation **(2)**) and excess vacancy concentration $c_{ex}$ with equation **(3)**. With the diffusion constant D (calculated according to **(4)**) and a diffusion enhancement factor f (equation **(5)**), the diffusion paths can be determined by **(6)**. Equations **(3)** and **(6)** were thereby solved iteratively with an explicit integration scheme[7].

$$\boldsymbol{c_0 = 2.3 \cdot \exp\left(-\frac{H_V^f}{RT}\right)} \text{ (2)}$$

$$\boldsymbol{\frac{dc_{ex}}{dt} = \chi \frac{\sigma \Omega_0}{H_V^f} \dot{\varepsilon} - \left(\frac{D_V \rho}{\kappa^2} + \frac{D_V}{L^2}\right) c_{\mathrm{ex}}} \text{ (3)}$$

$$\boldsymbol{D_{Mg,Si,Cu} = D^0_{Mg,Si,Cu} \cdot \exp\left(-\frac{H^d_{Mg,Si,Cu} - H_V^f}{RT}\right)} \text{ (4)}$$

$$\boldsymbol{f = 1 + \frac{c_{ex}}{c_0}} \text{ (5)}$$

$$\boldsymbol{x^2_{Mg,Zn,Cu} = \int 4 \cdot f \cdot D_{Mg,Zn,Cu} \cdot dt} \text{ (6)}$$

The effective diffusion in dependence of vacancy concentration and pipe-diffusion along dislocation cores can be expressed by **(7)**. For the pipe-diffusion-coefficient $D_p$ the activation energy for diffusion was assumed to be $0.55 \cdot H_j^d$ (activation energy for lattice diffusion)[6].

$$\boldsymbol{\mathrm{D}_{eff_{Mg,Si,Cu}} = D_{Mg,Si,Cu} \cdot \left(1 + \frac{c_{ex}}{c_0} + g \cdot \frac{D_{p_{Mg,Si,Cu}}}{D_{Mg,Si,Cu}}\right)} \text{ (7) with } \boldsymbol{g = 2 \cdot \Omega_0 \cdot \frac{\rho}{b}}$$

The dislocation enhancement factor is defined as $\left(1 + \frac{c_{ex}}{c_0} + g \cdot \frac{D_{p_{Mg,Si,Cu}}}{D_{Mg,Si,Cu}}\right)$.

$$c_0 = 2.3 \cdot \exp\left(-\frac{64{,}000\ \frac{\mathrm{J}}{\mathrm{mol}}}{8.314\ \frac{\mathrm{J}}{\mathrm{mol \cdot K}} \cdot 373\ \mathrm{K}}\right) = 2.51 \cdot 10^{-9} = 2.51 \cdot 10^{-3}\ ppm$$

$$\frac{c_{ex}}{c_0} = \frac{2 \cdot 10^3\ \mathrm{ppm}}{2.51 \cdot 10^{-3}\ \mathrm{ppm}} = 8\ \cdot 10^5$$

$$g = 2 \cdot\ 1.66\ \cdot 10^{-29}\ \mathrm{m}^3 \cdot \frac{3 \cdot 10^{14}\ \mathrm{m}^{-2}}{2.86 \cdot 10^{-10}\ \mathrm{m}} = 3.48 \cdot 10^{-5}$$

As $\frac{c_{ex}}{c_0}$ and $g$ are constant for Mg, Si and Cu, only the dislocation enhancement by dislocations (pipe-diffusion) has to be determined separately:

$$g \cdot \frac{D_{p_{Mg}}}{D_{Mg}} = 3.48 \cdot 10^{-5} \cdot \frac{D_{0_{Mg}} \cdot \exp^{-\frac{0.55 \cdot 115{,}000 \frac{\mathrm{J}}{\mathrm{mol}}}{8.314 \frac{\mathrm{J}}{\mathrm{mol \cdot K}} \cdot 298\ \mathrm{K}}}}{D_{0_{Mg}} \cdot \exp^{-\frac{115{,}000 \frac{\mathrm{J}}{\mathrm{mol}}}{8.314 \frac{\mathrm{J}}{\mathrm{mol \cdot K}} \cdot 298\ \mathrm{K}}}} = 4.09 \cdot 10^4$$

$$g \cdot \frac{D_{p_{Si}}}{D_{Si}} = 1.46 \cdot 10^5$$

$$g \cdot \frac{D_{p_{Cu}}}{D_{Cu}} = 1.55 \cdot 10^6$$

The combined diffusion enhancement factor (excess vacancies and dislocations) results $8.41 \cdot 10^5$ for Mg, $9.46 \cdot 10^5$ for Si and $2.35 \cdot 10^6$ for Cu.

The results for the calculations are shown in **Supplementary Fig. 10**, for a summary of the applied parameters see **Supplementary Table 4**.

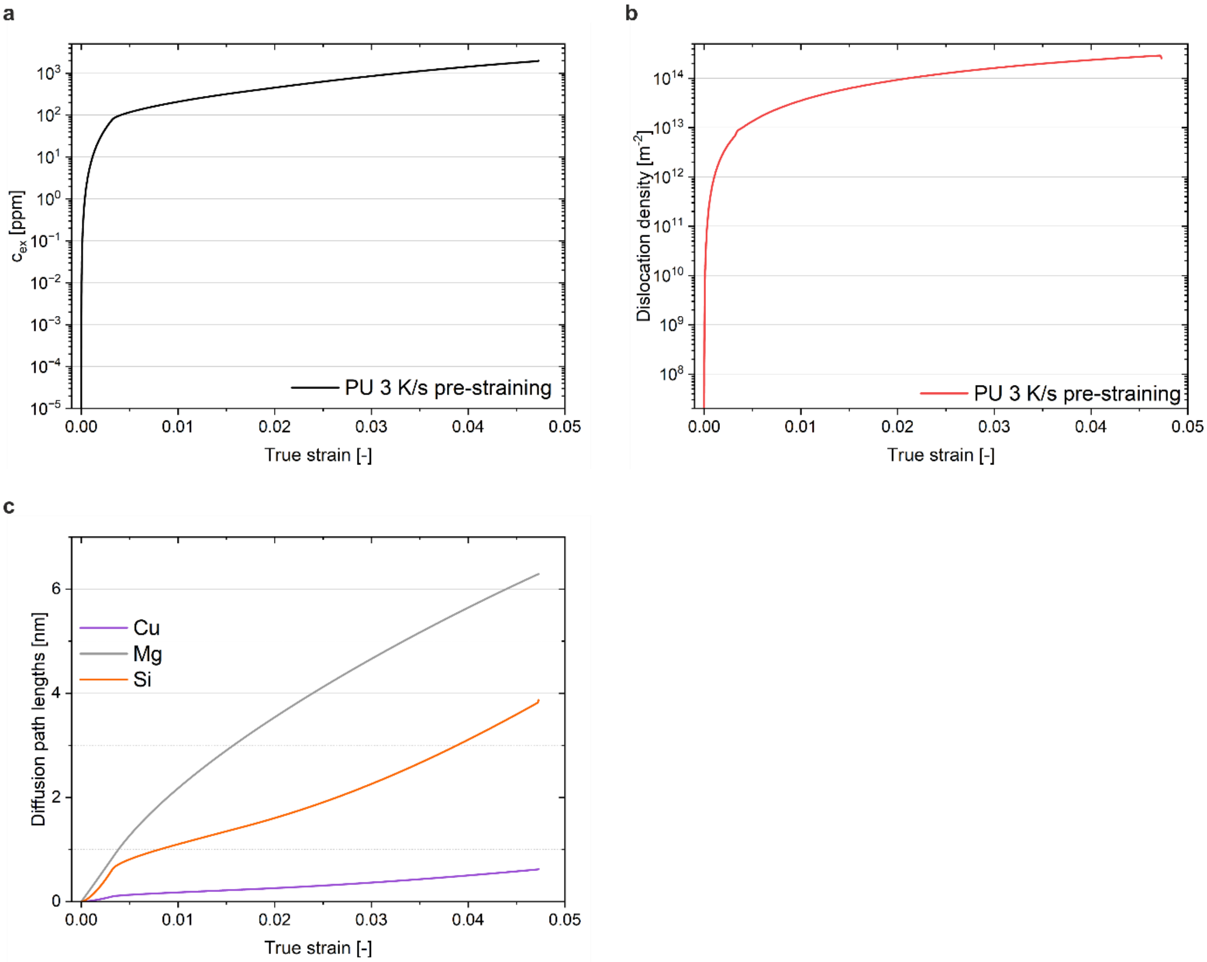


**Supplementary Fig. 10: Diffusion enhancement by pre-straining. a** Introduction of excess vacancies during pre-deformation. **b** Increase of the dislocation density during pre-deformation resulting in more favourable nucleation places. **c** Extension of diffusion paths for Cu, Mg and Si through pre-straining.

**Supplementary Table 4: Summary of the applied parameters for vacancy, dislocation density and diffusion path calculations.**

| Parameter | Notation | Value |
|---|---|---|
| Grain size–3 K/s | $d_{3\,K/s}$ | ~ 16 µm |
| Constant | $\alpha$ | 0.3 [8] |
| Taylor factor | $M$ | 3.06 [8] |
| Shear modulus | $G$ | 25.4 GPa [8] |
| Burgers vector | $b$ | $0.286 \cdot 10^{-9}$ m [8] |
| Fraction of external work stored by producing vacancies | $\chi$ | 0.1 [5,6] |
| Atomic volume | $\Omega_0$ | $1.66 \cdot 10^{-29}$ $m^3$ [6] |
| Vacancy / self-diffusion enthalpy | $H_V^{sd}$ | 129 $kJ \cdot mol^{-1}$ [9] |
| Vacancy formation enthalpy | $H_V^f$ | 64 $kJ \cdot mol^{-1}$ [9] |
| Vacancy migration enthalpy | $H_V^m$ | 65 $kJ \cdot mol^{-1}$ [6] |
| Mg diffusion / Activation enthalpy | $H_{Mg}^d$ | 115 $kJ \cdot mol^{-1}$ [10] |
| Si diffusion / Activation enthalpy | $H_{Zn}^d$ | 122 $kJ \cdot mol^{-1}$ [11] |
| Cu diffusion / Activation enthalpy | $H_{Cu}^d$ | 135 $kJ \cdot mol^{-1}$ [11] |
| Pre-factor vacancy diffusion | $D_V^0$ | $3 \cdot 10^{-5}$ $m^2 \cdot s^{-1}$ [6] |

| | | |
|---|---|---|
| Pre-factor Mg diffusion | $D_{Mg}^{0}$ | $6.23 \cdot 10^{-6}$ m$^{2} \cdot$s$^{-1}$ [10] |
| Pre-factor Si diffusion | $D_{Zn}^{0}$ | $1.19 \cdot 10^{-5}$ m$^{2} \cdot$s$^{-1}$ [11] |
| Pre-factor Cu diffusion | $D_{Cu}^{0}$ | $6.47 \cdot 10^{-5}$ m$^{2} \cdot$s$^{-1}$ [11] |
| Dislocation arrangement | $\kappa$ | 1.0 [5] |
| Gas constant | $R$ | 8.314 J·mol$^{-1} \cdot$K$^{-1}$ [12] |

**Supplementary References**


1. Krall, P., Weißensteiner, I. & Pogatscher, S. Recycling aluminum alloys for the automotive industry: Breaking the source-sink paradigm. *Resources, Conservation and Recycling* 202, 107370. 10.1016/j.resconrec.2023.107370 (2024).
2. Ansys (2025). *Ansys Granta Research Selector, Release 2025 R2*. Level 3 Aero database. Cambridge, UK: Ansys, Inc.
3. Ceguerra, A. V., Moody, M. P., Stephenson, L. T., Marceau, R. K. & Ringer, S. P. A three-dimensional Markov field approach for the analysis of atomic clustering in atom probe data. *Philosophical Magazine* 90, 1657–1683. 10.1080/14786430903441475 (2010).
4. Arnberg, L. *et al*. The Crystal Structure of Al(x)Cu2Mg(12-x)Si7, (h-AlCuMgSi). *Acta Chem. Scand*. 34a, 1–5. 10.3891/acta.chem.scand.34a-0001 (1980).
5. Militzer, M., Sun, W. P. & Jonas, J. J. Modelling the effect of deformation-induced vacancies on segregation and precipitation. *Acta Metallurgica et Materialia* 42, 133–141. 10.1016/0956-7151(94)90056-6 (1994).
6. Robson, J. D. Deformation Enhanced Diffusion in Aluminium Alloys. *MTA* 51, 5401–5413. 10.1007/s11661-020-05960-5 (2020).
7. Krall, P. *et al*. Direct aluminium-alloy upcycling from entire end-of life vehicles. 10.5281/zenodo.17192766 (2025).
8. Cheng, L. M., Poole, W. J., Embury, J. D. & Lloyd, D. J. The influence of precipitation on the work-hardening behavior of the aluminum alloys AA6111 and AA7030. *MTA* 34, 2473–2481. 10.1007/s11661-003-0007-2 (2003).
9. Mundy, J. N. Diffusion Mechanism in F.C.C. Metals. *Physica Status Solidi (b)* 144, 233–241. 10.1002/pssb.2221440121 (1987).
10. Fujikawa, S. & Hirano, K. Diffusion of 28Mg in aluminum. *Materials Science and Engineering* 27, 25–33. 10.1016/0025-5416(77)90190-2 (1977).
11. Czerwinski, F. Thermal Stability of Aluminum Alloys. *Materials (Basel, Switzerland)* 13. 10.3390/ma13153441 (2020).
12. Gottstein, G. *Materialwissenschaft und Werkstofftechnik*. Berlin, Heidelberg: Springer Berlin Heidelberg.
